# Natural Phenolic Compounds for Health, Food and Cosmetic Applications

**DOI:** 10.3390/antiox9050427

**Published:** 2020-05-15

**Authors:** Lucia Panzella

**Affiliations:** Department of Chemical Sciences, University of Naples “Federico II”, Via Cintia 4, I-80126 Naples, Italy; panzella@unina.it; Tel.: +39-081-674-131

Based on their potent antioxidant properties, natural phenolic compounds have gained more and more attention for their possible exploitation as food supplements, as well as functional ingredients in food and in the cosmetic industry [1,2,3,4,5,6,7,8,9]. This Special Issue, concerning innovative applications of natural phenolic compounds from edible or non-edible natural sources in the field of nutrition, biomedicine, food, and the cosmetic sector, contains fifteen contributions, of which fourteen are research articles and one review.

The phytochemical constituents, as well as the antioxidant, cytotoxic, and antimicrobial activities of the ethanolic extract of Mexican brown propolis have been reported by Rivero-Cruz et al. [10]. Twelve known compounds have been isolated and identified by nuclear magnetic resonance spectroscopy (NMR). Additionally, 40 volatile compounds, including nonanal, α-pinene, and neryl alcohol, have been identified by means of headspace-solid phase microextraction with gas chromatography and mass spectrometry time of flight analysis (HS-SPME/GC-MS-TOF). The extract showed anti-proliferative effects on glioma cells and was able to decrease the proliferation and viability of cervical cancer cells. 

Lin et al. [11] investigated the effect of isoliquiritigenin (ISL) on the proliferation of triple-negative breast cancer cells. The authors found that treatment with ISL inhibited triple-negative breast cancer cell line (MDA-MB-231) cell growth and increased cytotoxicity. ISL was able to reduce cell cycle progression through the reduction of cyclin D1 protein expression and increased the sub-G1 phase population. The expression of Bcl-2 protein was reduced by ISL treatment, whereas the Bax protein level increased; subsequently, the downstream signaling molecules caspase-3 and poly ADP-ribose polymerase (PARP) were activated. Moreover, ISL reduced the expression of total and phosphorylated mammalian target of rapamycin (mTOR), ULK1, and cathepsin B, whereas the expression of autophagic-associated proteins p62, Beclin1, and LC3 was increased. In vivo studies further confirmed that preventive treatment with ISL could inhibit breast cancer growth and induce apoptotic and autophagic-mediated apoptosis cell death. 

Natural phenolic compound rich-extracts have also been recently described as effective agents against environmental oxidative stressors, such as mercury. In particular, Tortora et al. [12] reported the beneficial properties of *Feijoa sellowiana* extracts against mercury toxicity in human red blood cells (RBCs). Both peel and pulp extracts were able to counteract the oxidative stress and thiol decrease induced in RBCs by mercury treatment, although the peel extract had a greater protective effect due in part to the amount and kind of phenolic compounds. Furthermore, *Fejioa sellowiana* extracts also prevented mercury-induced morphological changes, which are known to enhance the pro-coagulant activity of RBCs. 

Increasing attention has also been recently devoted to the development of formulations allowing for higher stability and bioavailability of bioactive phenols. In particular, Shimojo et al. [13] have reported optimization of the production process of nanostructured lipid carriers (NLCs) for resveratrol. NLCs were produced by a high shear homogenization and ultrasound method using Compritol^®^ ATO C888 as a solid lipid and Miglyol 812^®^ as a liquid lipid. Based on the factorial design, which was used to optimize the variables of the NLCs production process from a small number of experiments, it was concluded that a shear rate of 19,000 rpm and a shear time of 6 min was the optimal parameters for resveratrol-loaded NLC production. 

Along the same line, Ha et al. [14] created composite nanoparticles containing hydrophilic additives using a supercritical antisolvent (SAS) process to increase the solubility and dissolution properties of resveratrol for application in oral and skin delivery. In particular, resveratrol/hydroxylpropylmethyl cellulose (HPMC)/poloxamer 407 (1:4:1) nanoparticles with the highest flux (0.792 μg/min/cm^2^) exhibited rapid absorption and showed significantly higher exposure 4 h after oral administration, compared to micronized resveratrol. Good correlations were observed between in vitro flux and in vivo pharmacokinetic data. The increased solubility and flux of resveratrol generated by the HPMC/surfactant nanoparticles increased the driving force on the gastrointestinal epithelial membrane and rat skin, resulting in enhanced oral and skin delivery of the compound. 

Gelatin-based hydrogels have instead been reported for the controlled release of 5,6-dihydroxyindole-2-carboxylic acid (DHICA), a melanin-related metabolite with potent antioxidant activity. In particular, a paper by Alfieri et al. [15] describes the preparation of three types of gelatin-based hydrogels, that is a pristine porcine skin type A gelatin (HGel-A), a pristine gelatin cross-linked by amide coupling of lysines and glutamic/aspartic acids (HGel-B), and a gelatin/chitosan blend (HGel-C). The extent of incorporation into all the gelatins tested using a 10% *w*/*w* indole to gelatin ratio was very satisfactory, ranging from 60 to 90%, and an appreciable release under conditions of physiological relevance was observed, reaching 30% and 40% at 6 h for HGel-B and HGel-C, respectively. Moreover, DHICA incorporated into HGel-B proved fairly stable over 6 h, whereas the free compound at the same concentration was almost completely oxidized. The antioxidant power of the indole loaded gelatins was also monitored by chemical assays and proved unaltered even after prolonged storage in air.

The potent photoprotective and antioxidant activities of a DHICA-related phenolic polymer have also been reported by Liberti et al. [16]. In particular, the protective effect of a polymer obtained starting from the methyl ester of DHICA (MeDHICA-melanin) against ultraviolet A (UVA)-induced oxidative stress in immortalized human keratinocytes (HaCaTs) was described. At concentrations as low as 10 µg/mL, MeDHICA-melanin prevented reactive oxygen species accumulation and partially reduced glutathione oxidation in UVA-irradiated keratinocytes. Western blot experiments revealed that the polymer was able to induce the translocation of nuclear factor erythroid 2-related factor 2 (Nrf-2) to the nucleus with the activation of the transcription of antioxidant enzymes, such as heme-oxygenase 1. Spectrophotometric and HPLC analysis of cell lysate allowed the conclusion that a significant fraction (ca. 7%) of the polymer was internalized in the cells.

Instability issues concerning resveratrol were the object of investigation in a paper by Leyva-Porras et al. [17], who studied the effect of the spray drying processing conditions of blueberry juice and maltodextrin (MX) mixtures on the content and retention of resveratrol in the final product. Analysis of variance (ANOVA) showed that the concentration of MX was the main variable influencing resveratrol content. Response surface plots (RSP) confirmed the application limits of maltodextrins based on their molecular weight, where low molecular weight MXs showed better performance as carrying agents. 

López de Dicastillo et al. [18] instead reported the encapsulation of açaí fruit antioxidants, especially anthocyanins, into electrosprayed zein, a heat-resistant protein, to improve their bioavailability and thermal resistance. In particular, a hydroalcoholic açaí extract was selected due to its high polyphenolic content and antioxidant capacities. Encapsulation efficiency was approximately 70%. Results demonstrated the effectiveness of the encapsulation on protecting polyphenolic content after high-temperature treatments, such as sterilization and baking. Bioaccessibility studies also indicated an increase of polyphenol levels after in vitro digestion stages of encapsulated açaí fruit extract in contrast with the unprotected extract.

Bioactive compound nanoemulsions have been evaluated as coatings to improve avocado fruit quality during postharvest by Cenobio-Galindo et al. [19]. Nanoemulsions made of orange essential oil and *Opuntia oligacantha* extract were applied as a coating in whole avocado fruits, and the following treatments were assessed: concentrated nanoemulsion (CN), 50% nanoemulsion (N50), 25% nanoemulsion (N25) and control (C). The best results were obtained with the N50 and N25 treatments not only for firmness and weight loss but also for the activity of polyphenol oxidase since a delay in browning was observed in the coated fruits. Furthermore, the nanoemulsion treatments maintained the total phenol and total flavonoid content and improved the antioxidant activity as determined by 2,2′-azino-bis(3-ethylbenzothiazoline-6-sulfonic acid) (ABTS) and 2,2-diphenyl-1-picrylhydrazyl (DPPH) assays at 60 days compared to controls. A delaying effect on the maturation of the epicarp was also observed when the nanoemulsion was applied. 

Puffing has instead been proposed to enhance ginsenoside content and antioxidant activities of ginseng (*Panax quinquefolius*). In particular, Kim et al. [20] analyzed American and Canadian ginsengs puffed at different pressures and extracted with 70% ethanol. Puffing formed a porous structure, inducing an efficient elution of internal compounds that resulted in significant increases in extraction yields. The content of minor ginsenosides such as Rg2, Rg3, and compound K increased with increasing puffing pressure, whereas that of the major ginsenosides Rg1, Re, Rf, Rb1, Rc, Rd decreased, likely as a consequence of deglycosylation and pyrolysis processes. ABTS radical scavenging activity, total phenolic content, and total flavonoid content increased with increasing puffing pressure, although no improvement in DPPH reducing activity was observed. 

Haydari et al. [21] reported that the natural additives salicylic acid and melatonin were able to alleviate the effects of heat stress on essential oil composition and antioxidant enzyme activity (catalase, superoxide dismutase, glutathione S-transferase, and peroxidase) in *Mentha piperita* and *Mentha arvensis* L. 

Natural phenolic compounds are widely present not only in foods but also in non-edible, easily-accessible sources, such as waste materials from agri-food industries [22]. In this context, Panzella et al. [23] focused their attention on exhausted woods, which represent a by-product of the tannin industrial production processes. In particular, the authors reported the characterization of the antioxidant and other properties of practical interest of exhausted chestnut and quebracho wood, together with those of a chestnut wood fiber, produced from steamed exhausted chestnut wood. All the materials investigated exhibited good antioxidant properties in DPPH and ferric reducing/antioxidant power (FRAP) assays, as well as in a superoxide scavenging assay. An increase of the antioxidant potency was observed for both exhausted woods and chestnut wood fiber following activation by hydrolytic treatment. The three materials also proved able to adsorb organic pollutants and to remove toxic heavy metal ions from aqueous solutions. 

In recent years, particular attention has also been devoted to modulation of the solubility properties of natural phenolic compounds to broaden their applications, e.g., as dietary supplements or stabilizers of foods and cosmetics in non-aqueous media. In this regard, Bernini et al. [24] reported a simple and low-cost procedure for the synthesis of lipophilic esters of tyrosol, homovanillyl alcohol, and hydroxytyrosol. The reactions were carried out under mild and green chemistry conditions, which also allowed obtaining the desired products in good yields. Notably, the procedure was also applied to hydroxytyrosol-enriched extracts obtained from olive by-products.

Finally, the cellular, antioxidant, and anti-inflammatory properties of cannabidiol and its synthetic derivatives have been reviewed by Atalay et al. [25].

In conclusion, the contributions published in this Special Issue open new perspectives toward the exploitation of phenol-rich natural extracts or pure phenolic compounds as functional ingredients in the health sector, e.g., in preventing and combating mercury-related illnesses or as alternative therapeutic agents for clinical trials against breast cancer. The growing importance of agri-food wastes as sources of phenolic compounds, as well as of synthetic derivatives of natural compounds with improved antioxidant properties have also been highlighted. Finally, novel technologies have been described to improve extraction yields, stability, bioavailability, and delivery of antioxidant compounds, e.g., for healthcare products or for skin applications.
